# Plasma Metabolomics Reveals Diagnostic Biomarkers and Risk Factors for Esophageal Squamous Cell Carcinoma

**DOI:** 10.3389/fonc.2022.829350

**Published:** 2022-02-07

**Authors:** Mengjie Yu, Wei Wen, Xin Yi, Wei Zhu, Jiye Aa, Guangji Wang

**Affiliations:** ^1^ Key Laboratory of Drug Metabolism and Pharmacokinetics, China Pharmaceutical University, Nanjing, China; ^2^ Department of Thoracic Surgery, First Affiliated Hospital of Nanjing Medical University, Nanjing, China; ^3^ Department of Oncology, First Affiliated Hospital of Nanjing Medical University, Nanjing, China

**Keywords:** ESCC (esophageal squamous cell carcinoma), metabolomics, biomarker, risk factors, esophageal squamous dysplasia

## Abstract

Esophageal squamous carcinoma (ESCC) has a high morbidity and mortality rate. Identifying risk metabolites associated with its progression is essential for the early prevention and treatment of ESCC. A total of 373 ESCC, 40 esophageal squamous dysplasia (ESD), and 218 healthy controls (HC) subjects were enrolled in this study. Gas chromatography-mass spectrometry (GC/MS) was used to acquire plasma metabolic profiles. Receiver operating characteristic curve (ROC) and adjusted odds ratio (OR) were calculated to evaluate the potential diagnosis and prediction ability markers. The levels of alpha-tocopherol and cysteine were progressively decreased, while the levels of aminomalonic acid were progressively increased during the various stages (from precancerous lesions to advanced-stage) of exacerbation in ESCC patients. Alpha-tocopherol performed well for the differential diagnosis of HC and ESD/ESCC (AUROC>0.90). OR calculations showed that a high level of aminomalonic acid was not only a risk factor for further development of ESD to ESCC (OR>13.0) but also a risk factor for lymphatic metastasis in ESCC patients (OR>3.0). A low level of alpha-tocopherol was a distinguished independent risk factor of ESCC (OR< 0.5). The panel constructed by glycolic acid, oxalic acid, glyceric acid, malate and alpha-tocopherol performed well in distinguishing between ESD/ESCC from HC in the training and validation set (AUROC>0.95). In conclusion, the oxidative stress function was impaired in ESCC patients, and improving the body’s antioxidant function may help reduce the early occurrence of ESCC.

## Introduction

Esophageal cancer (EC) is the seventh most common cancer and the sixth most common cause of cancer death globally, causing about 572 000 new cases and 509 000 deaths worldwide (1). Esophageal squamous cell carcinomas (ESCC) are the most common histological type of EC, accounting for approximately 90% of esophageal cancer cases worldwide (2). China has the highest incidence of ESCC, accounting for approximately 50% of all ESCC cases worldwide (3, 4). ESCC has no specific clinical symptoms in its early stages, and most patients are diagnosed at an advanced stage, resulting in a 5-year survival rate of less than 15% (5). Esophageal squamous dysplasia (ESD) is a primary precancerous lesion for ESCC, with a significantly increased risk of developing into ESCC (6). Although endoscopy and histopathological testing can effectively improve the early diagnosis of ESCC (7, 8), these two methods are invasive and require trained physicians and expensive equipment, making them challenging to use widely in the early screening of ESCC. Therefore, surveying the metabolic change and associated risk factors occurring during ESCC and establishing suitable non-invasive adjunctive assays development could provide implications for early diagnosis and potential therapeutic strategies.

With its powerful screening and identification of small molecule metabolites, Metabolomics has become a powerful tool to identify metabolic changes in cancer progression and discover non-invasive biomarkers for cancer prediction and diagnosis (9–12). Currently, based on nuclear magnetic resonance (NMR) (13), gas chromatography-mass spectrometry (GC-MS) (14), liquid chromatography-mass spectrometry (LC-MS) (15), and other metabolomics techniques have been widely used in studies related to ESCC. Many non-invasive auxiliary essays related to ESCC have been established through plasma (16), serum (17) and urine (18). However, these studies have mainly focused on the role of small molecule metabolites in the progression of healthy controls (HC) and ESCC patients. Less attention has been paid to screening metabolic changes and associated risk factors during the progression of ESCC from early to advanced stages.

In this work, a two-phase development strategy (training set and validation set) was applied in 631 subjects, including clinically relevant controls covering the whole progression of ESCC. Based on the GC/MS metabolomics platform, we propose establishing a suitable non-invasive diagnostic approach and screening for risk factors associated with ESCC progression. This work could help discover new biomarkers for risk prediction and early detection of ESCC.

## Materials and Methods

### Chemicals and Reagents

1, 2-^13^C_2_-Myristic acid and methyl myristate were used as internal standard (IS) and external standard (ES), respectively. 1, 2-^13^C_2_-Myristic acid, methyl myristate, methoxyamine, MSTFA (N-methyl-N-trimethylsilyltrifluoroacetamid) plus 1% TMCS, n-heptane, and pyridine (silylation grade) were obtained from Sigma-Aldrich. HPLC-grade (>99.5%) methanol was obtained from Merck.

### Sample Pretreatment

Plasma samples were processed, extracted, and derived following our previously developed methods (19, 20). An aliquot of plasma (50 µL) was added to 200 µl methanol (containing IS, 5.0 µg/mL). The specimens were vigorously extracted for 5.0 min and centrifuged at 20 000×g for 10.0 min at 4°C. A 100.0 μL aliquot of the resulting supernatant was transferred to a GC vial and evaporated to dryness in a Speed-Vac concentrator (Savant Instruments, Farmingdale, NY, USA). 30.0 μL of methoxyamine in pyridine (10.0 mg/mL) was added to each GC vial. Then the solution was vigorously vortexed for 5.0 min. After methoximation reaction for 16.0 hours at room temperature, the samples were trimethylsilylated for another 1.0 hours by adding 30.0 μL of MSTFA with 1% TMCS as the catalyst. At last, 30.0 μL n-heptane with methyl myristate (15.0 µg/mL) as the quality control reference standard was added to each GC vial. The quality control samples (QC) were pooled with small aliquots of plasma samples in the study set and mixed.

### GC/MS Analysis, Instrumental Setting, and Parameters

To diminish the opportunity for systematic variation, all the samples were randomly selected for analysis by GC/MS. A 0.5 μL portion of the derived samples was injected into Shimadzu GC/MS QP2010Ultra/SE (Kyoto, Japan). It is equipped with a 30 m × 0.25 mm ID, fused silica capillary column, which was chemically bonded with 0.25 m DB1-MS stationary phase (J&W Scientific, Folsom, CA, USA).

The column temperature was initially kept at 80°C for 3.0 min, then increased from 80 to 300°C at 20°C/min, where it was held for 5.0 min. The transfer line temperature was set at 220°C and the ion source temperature at 200°C. Ions were generated by a 70-eV electron beam at a current of 3.2 mA. The mass spectra were acquired over the mass range of 50-700 m/z at a rate of 25 spectra/s after a solvent delay of 160 s.

The metabolites were by comparing the mass spectrum and retention indexes for the analyte with the corresponding values from the literature and various libraries [e.g., Mainlib and Public in the National Institute of Standards and Technology (NIST) library 2.0 (2008) and Wiley 9 (Wiley-VCH Verlag GmbH & Co KGaA, Weinheim, Germany)].

### Statistical Analysis

After normalization against the IS, all the semiquantitative data were log_10_-transformed. The transformed data were imported into SIMCA-P 14.1 software (Umetrics, Umea, Sweden) and pre-processed for multivariate statistical analysis using unit variance scaling (UV). Principal component analysis (PCA) and orthogonal projections to latent structures discriminant analysis (OPLS-DA) models were built and plotted to show the clustering or separation of samples from different groups. The goodness of fit for the OPLS-DA models was evaluated using three quantitative parameters: R^2^X, R^2^Y and Q^2^. R^2^X and R^2^Y are the explained variations, and Q^2^ is the predicted variation, with a higher level of R^2^Y and Q^2^Y indicating the model’s better fit and predictive performance (21). To avoid the classification obtained by supervised learning methods being chance and to test whether the model reproduces well and whether the data in the model are over-fitted, the validity of the built model was examined by 7-fold cross-check and replacement test (200 times, cross-validation). The intercept of the R^2^ and Q^2^ regression lines to the axes was used to measure overfitting, and the model was valid when the intercept of Q^2^ was negative (22).

To determine the difference between groups, the independent-samples *t*-test and the Mann-Whitney U test were applied for normally and non-normally distributed data, respectively. The diagnostic performance of each metabolite was evaluated by the receiver operating characteristic (ROC) curve. The Youden index was the best threshold to select the optimal cut-point that maximized its value (23).

Metabolite variability analysis, logistic regression analysis, ROC curve analysis and (adjusted) OR calculations were performed using SPSS 26.0 (SPSS Inc., Chicago, IL, USA), bar graphs were produced using GraphPad Prism 8.0, and heatmap and pathway analysis were performed using the online software MetaboAnalyst (https://www.metaboanalyst.ca/).

## Results

### Patients and Healthy Controls

Samples for this study were collected at the First Affiliated Hospital of Nanjing Medical University, and the sampling period was from June 2019 to June 2021. Blood samples were collected before 8:30 am after overnight fasting to eliminate the disturbance of diet, and samples were kept under 4°C temperature before being stored at –80°C within 6 hours after plasma isolation (24). A total of 631 subjects were included in this study, including 218 healthy controls (HC), 373 with esophageal squamous cell carcinoma (ESCC) and 40 with esophageal squamous dysplasia (ESD). The distribution of subjects is shown in **Table 1**. We set stage 0 and stage I as early-stage, stage II and stage III as intermediate-stage, and stage IV as advanced-stage, taking into account the progression of ESCC and cTNM staging.

**Table 1:** Basic information of all subjects.

	Training set			Validation set		
	ESCC	ESD	HC	ESCC	ESD	HC
Number	346	20	187	27	20	31
Male/female	266/80	14/6	121/66	24/3	11/9	23/8
Age (years)	62.99 ± 11.94	58.10 ± 6.84	63.97 ± 6.88	60.52 ± 6.86	61.90 ± 7.67	61.22 ± 6.98
cTNM classification						
Stage 0	3			0		
Stage I	84			7		
Stage II	120			9		
Stage III	115			7		
Stage IV	24			4		
N-Regional Lymph Nodes						
N0	207			16		
N1	88			5		
N2	47			2		
N3	4			4		

**Figure 1:**
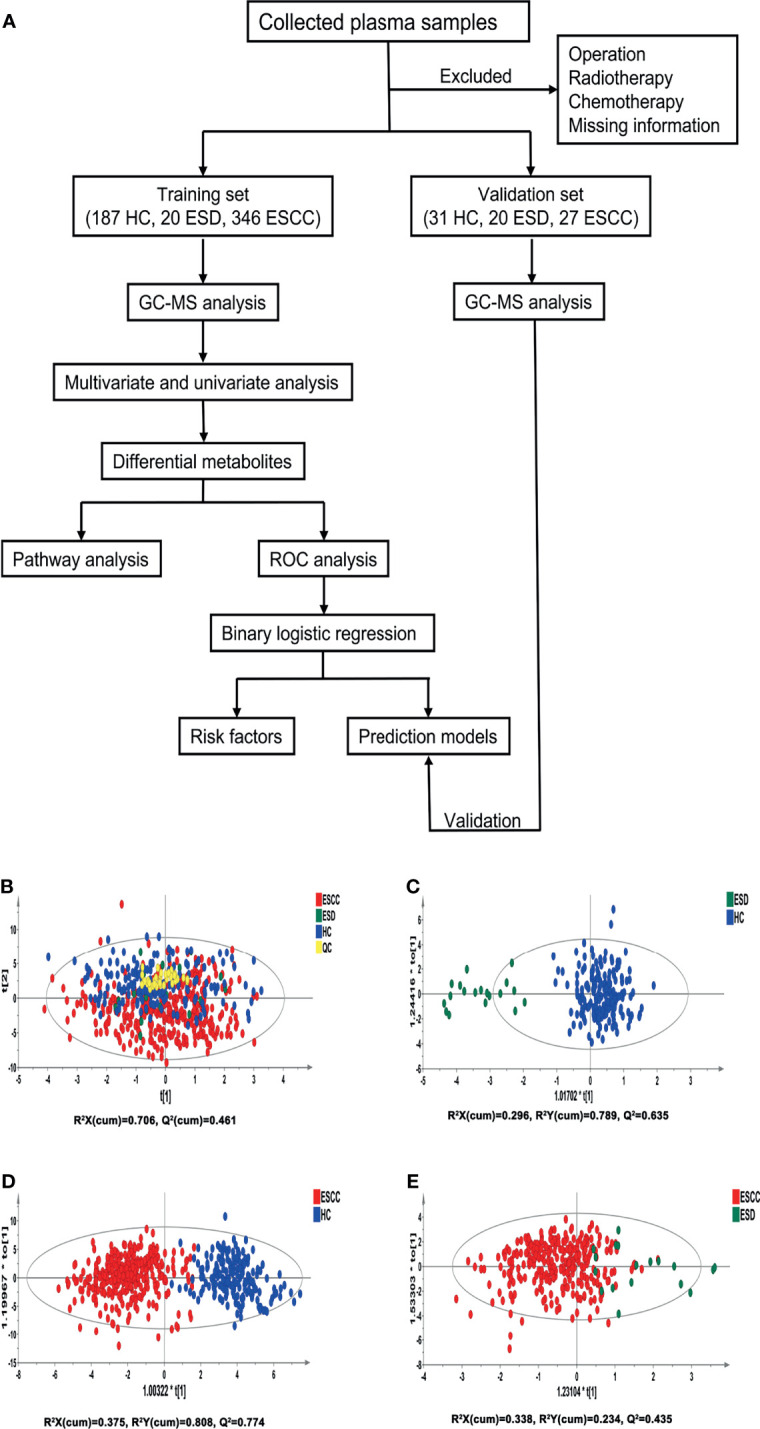
Analysis flowchart of this study and multivariate statistical analysis differentiates the groups of HC, ESD and ESCC. **(A)** Analysis flowchart of this study. **(B)** PCA modeling with the three groups: HC, ESD and ESCC. **(C)** OPLS-DA model differentiating ESD from HC. **(D)** OPLS-DA model differentiating ESCC from HC. **(E)** OPLS-DA model differentiating ESCC from ESD.

Subjects included in this study were free of metabolic abnormalities such as hypoproteinemia, weight loss, and negative nitrogen balance. The ethics committee of the First Affiliated Hospital of Nanjing Medical University approved this study, and informed consent was obtained from all subjects. The study flowchart is shown in **Figure 1A**.

### Clustering Analysis

Pooled QC samples were clustered well in the PCA score plots (**Figure 1B**), indicating stable instrument operation and good reproducibility of the assay throughout the experiment. The supervised OPLS-DA models revealed that the samples in the HC and ESD/ESCC groups were closely clustered together, with fewer overlapping areas between the groups (**Figures 1C, D**), indicating significant metabolic differences between the HC and ESD/ESCC groups. At the same time, the parameters of the two OPLS-DA models mentioned above were R^2^X=0.296, R^2^Y=0.789, Q^2 =^ 0.635 and R^2^X=0.375, R^2^Y=0.808, Q^2 =^ 0.774, respectively, indicating that the models had good fit and prediction accuracy. There was a partial overlap region between the ESD and ESCC groups (**Figure 1E**), indicating some similarity of metabolic phenotypes between the ESD and ESCC groups (R^2^X=0.338, R^2^Y=0.234, Q^2 =^ 0.435). The permutation test results showed that the intercept of Q^2^ was negative in all groups (**Supplementary Figures 1A-C**), indicating that our OPLS-DA models were not over-fitted and the models were valid. These results indicated significant differences in metabolic patterns between the HC and ESD groups or the HC and ESCC groups. At the same time, there were some similarities in the metabolic changes between the ESD and ESCC groups.

### Metabolic Difference Analysis

GC/MS analysis of the plasma samples aligned the metabolites in typical chromatograms (**Supplementary Figure 1**). Deconvolution of the GC/MS chromatograms produced 135 independent peaks from the plasma samples, 57 of which were authentically identified as metabolites (**Supplementary Table 1**). Quantitative data were acquired for each metabolite in the plasma samples of the HC, ESD and ESCC cases.

**Figure 2:**
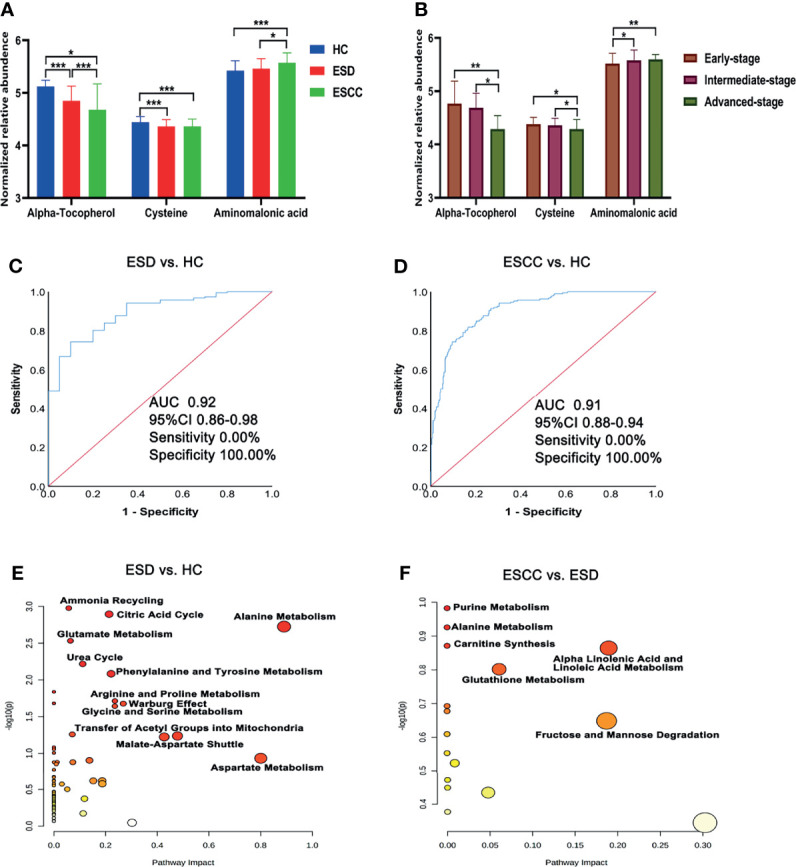
Differential metabolites and pathways involved in the ESD and ESCC groups. **(A)** Plasma alpha-tocopherol, cysteine, and aminomalonic acid levels in the HC, ESD and ESCC groups. **(B)** Plasma alpha-tocopherol, cysteine, and aminomalonic acid levels in different stages of ESCC (relative abundance is shown in logarized form: mean with SD, *p < 0.05, **0.001≤P < 0.01, ***P < 0.001). **(C)** ROC analysis of alpha-tocopherol between ESD and HC. **(D)** ROC analysis of alpha-tocopherol between ESCC and HC. **(E)** Pathway analysis of differential metabolites in ESD and HC. **(F)** Pathway analysis of differential metabolites in ESCC and ESD.

There were 35, 46, and 9 differential metabolites among HC, ESD, and ESCC groups (**Table 2**), and 3, 6, and 4 differential metabolites among early-stage, intermediate-stage, and advanced-stage groups, respectively (**Table 3**). Changes in the levels of three metabolites, alpha-tocopherol, aminomalonic acid and cysteine, correlated with the continuous progression of disease in ESCC patients. The levels of alpha-tocopherol and cysteine gradually decreased and the levels of aminomalonic acid gradually increased as the disease progressed in ESCC patients (**Figures 2A, B**). These findings indicate that the above metabolites are involved in the development of ESCC (from precancerous lesions to advanced-stage).

**Table 2:** List of discriminant metabolites: ESD vs. HC, ESCC vs. HC and ESCC vs. ESD.

Metabolites	HC (n=187)	ESD (n=20)	ESCC (n=346)	ESD vs. HC		ESCC vs. HC		ESCC vs. ESD	
				Trend	P	Trend	P	Trend	P
Glyceric acid	3.92 ± 0.08	4.05 ± 0.16	4.00 ± 0.18	up	0.002	up	0.000		/
Oxalic acid	5.38 ± 0.16	5.50 ± 0.11	5.54 ± 0.16	up	0.001	up	0.000		/
Hexadecanoic acid	3.18 ± 0.21	3.38 ± 0.20	3.42 ± 0.23	up	0.000	up	0.000		/
4-Hydroxybutanoic acid	4.82 ± 0.20	4.93 ± 0.17	4.85 ± 0.15	up	0.026	up	0.045	down	0.030
Nonanoic acid	3.71 ± 0.30	3.90 ± 0.28	3.74 ± 0.29	up	0.007		/	down	0.019
Arachidonic acid	4.23 ± 0.19	4.40 ± 0.21	4.25 ± 0.19	up	0.000		/	down	0.001
Glutamate	5.28 ± 0.19	5.16 ± 0.24	5.25 ± 0.22	down	0.005	down	0.033		/
Lysine	4.52 ± 0.20	4.43 ± 0.14	4.46 ± 0.17	down	0.033	down	0.000		/
Serine	5.51 ± 0.19	5.40 ± 0.15	5.44 ± 0.12	down	0.012	down	0.000		/
Fumarate	4.14 ± 0.17	4.03 ± 0.14	4.07 ± 0.17	down	0.003	down	0.000		/
Leucine	5.91 ± 0.16	5.84 ± 0.08	5.85 ± 0.11	down	0.002	down	0.000		/
Phenylalanine	5.22 ± 0.12	5.16 ± 0.06	5.17 ± 0.11	down	0.001	down	0.000		/
Aspartate	4.36 ± 0.15	4.19 ± 0.22	4.28 ± 0.23	down	0.003	down	0.000		/
Lactate	6.40 ± 0.19	6.25 ± 0.20	6.29 ± 0.32	down	0.001	down	0.000		/
Proline	5.64 ± 0.33	5.35 ± 0.47	5.49 ± 0.31	down	0.014	down	0.000		/
Valine	5.32 ± 0.15	5.22 ± 0.08	5.24 ± 0.11	down	0.000	down	0.000		/
Tyrosine	5.58 ± 0.13	5.50 ± 0.08	5.50 ± 0.12	down	0.000	down	0.000		/
Alanine	6.34 ± 0.20	6.19 ± 0.17	6.23 ± 0.16	down	0.001	down	0.000		/
Ornithine	5.00 ± 0.22	4.75 ± 0.25	4.85 ± 0.23	down	0.000	down	0.000		/
Citrate	5.70 ± 0.09	5.62 ± 0.10	5.64 ± 0.10	down	0.000	down	0.000		/
Cysteine	4.44 ± 0.11	4.36 ± 0.13	4.36 ± 0.14	down	0.001	down	0.000		/
Myo-Inositol	4.75 ± 0.12	4.66 ± 0.09	4.67 ± 0.12	down	0.001	down	0.000		/
Pyruvate	4.66 ± 0.16	4.44 ± 0.33	4.46 ± 0.38	down	0.007	down	0.000		/
Succinate	3.79 ± 0.14	3.69 ± 0.18	3.66 ± 0.17	down	0.002	down	0.000		/
Pyrophosphoric acid	5.10 ± 0.11	4.96 ± 0.10	4.99 ± 0.14	down	0.000	down	0.000		/
Asparagine	4.42 ± 0.17	4.23 ± 0.13	4.26 ± 0.14	down	0.000	down	0.000		/
Glutamine	6.05 ± 0.13	5.89 ± 0.10	5.92 ± 0.12	down	0.000	down	0.000		/
Palmitic acid	5.72 ± 0.09	5.61 ± 0.11	5.61 ± 0.13	down	0.000	down	0.000		/
Monopalmitin	4.32 ± 0.10	4.24 ± 0.11	4.20 ± 0.11	down	0.001	down	0.000		/
Malate	4.00 ± 0.10	3.82 ± 0.21	3.82 ± 0.22	down	0.001	down	0.000		/
Linoleic acid	5.16 ± 0.12	4.97 ± 0.16	4.97 ± 0.19	down	0.000	down	0.000		/
Uric acid	5.81 ± 0.15	5.65 ± 0.16	5.74 ± 0.18	down	0.000	down	0.000	up	0.032
Alpha-Tocopherol	5.12 ± 0.12	4.85 ± 0.28	4.68 ± 0.49	down	0.000	down	0.000	down	0.019
Fructose	4.65 ± 0.51	4.29 ± 0.54	4.59 ± 0.52	down	0.003		/	up	0.013
Glycine	4.23 ± 0.40	3.97 ± 0.14	4.09 ± 0.27	down	0.000	down	0.000	up	0.001
Cholesterol	5.86 ± 0.07	5.69 ± 0.66	5.83 ± 0.20		/	down	0.048		/
Urea	5.34 ± 0.34	5.39 ± 0.29	5.41 ± 0.36		/	up	0.042		/
Threonine	5.14 ± 0.17	5.10 ± 0.13	5.10 ± 0.12		/	down	0.015		/
3-Hydroxybutyric acid	4.86 ± 0.33	4.76 ± 0.37	4.77 ± 0.41		/	down	0.005		/
Fructose-6-Phosphate	3.88 ± 0.14	3.87 ± 0.12	3.84 ± 0.13		/	down	0.005		/
Oleic acid	5.56 ± 0.16	5.49 ± 0.15	5.51 ± 0.14		/	down	0.001		/
Monomethylphosphate	4.53 ± 0.14	4.47 ± 0.10	4.49 ± 0.12		/	down	0.001		/
Palmitelaidic acid	4.11 ± 0.26	3.95 ± 0.35	4.02 ± 0.31		/	down	0.001		/
Glucose	5.89 ± 0.28	6.03 ± 0.60	6.04 ± 0.39		/	up	0.000		/
Pyroglutamate	5.63 ± 0.07	5.54 ± 0.22	5.56 ± 0.21		/	down	0.000		/
Methionine	4.72 ± 0.14	4.66 ± 0.10	4.65 ± 0.11		/	down	0.000		/
Glycolic acid	3.86 ± 0.10	3.91 ± 0.21	3.92 ± 0.14		/	up	0.000		/
Aminomalonic acid	5.42 ± 0.19	5.46 ± 0.19	5.57 ± 0.19		/	up	0.000	up	0.013
Creatinine	4.47 ± 0.20	4.50 ± 0.17	4.61 ± 0.16		/	up	0.000	up	0.004

The data were log10 transformed and expressed as mean ± SD. “/” represents the statistical significance of p-values more than 0.05.

**Table 3:** List of discriminant metabolites: intermediate-stage vs. early-stage, advanced-stage vs. early-stage and advanced-stage vs. intermediate-stage.

Metabolites	Early-stage (n=87)	Intermediate-stage (n=235)	Advanced-stage (n=24)	Intermediate-stage vs. Early-stage		Advanced-stage vs. Early-stage		Advanced-stage vs. Intermediate-stage	
				Trend	P	Trend	P	Trend	P
Aminomalonic acid	5.52 ± 0.19	5.58 ± 0.19	5.60 ± 0.09	up	0.027	up	0.006		/
Aspartate	4.23 ± 0.24	4.29 ± 0.22	4.33 ± 0.22	up	0.049		/		/
Glutamate	5.20 ± 0.24	5.26 ± 0.22	5.28 ± 0.17	up	0.023		/		/
Pyrophosphoric acid	4.99 ± 0.14	4.98 ± 0.13	5.06 ± 0.15		/	up	0.020	up	0.003
Cysteine	4.38 ± 0.13	4.36 ± 0.13	4.29 ± 0.18		/	down	0.022	down	0.047
Arachidonic acid	4.26 ± 0.19	4.26 ± 0.19	4.14 ± 0.16		/	down	0.007	down	0.003
Alpha-Tocopherol	4.77 ± 0.42	4.69 ± 0.47	4.29 ± 0.75		/	down	0.006	down	0.019
Uric acid	5.72 ± 0.20	5.75 ± 0.17	5.81 ± 0.16		/	up	0.038		/

ROC analysis showed (**Supplementary Tables 2**–**4** and **Figures 2C, D**) that alpha-tocopherol performed well for the differentiation of HC and ESD/ESCC (AUROC>0.90). This suggests that alpha-tocopherol may be a diagnostic biomarker for the differentiation of HC and ESD/ESCC. However, for the differentiation of ESD and ESCC, each metabolite performed poorly (AUROC<0.72).

Metabolic pathway analysis (**Figures 2E, F**) showed that the HC and ESD groups were affected mainly by amino acid metabolism (urea cycle, glutamate metabolism, arginine and proline metabolism, etc.) and energy metabolism (citric acid cycle and Warburg effect). Metabolic pathways such as purine metabolism, alanine metabolism and carnitine synthesis may be further affected as ESD progresses to ESCC.

### Risk Metabolites Associated With ESCC

To assess the role of the above metabolites as risk factors for predicting ESD/ESCC occurrence, OR values were calculated. Plasma glyceric acid, oxalic acid, hexadecanoic acid and 4-hydroxybutanoic acid were all had ORs > 1 (ESD vs. HC) (**Table 4**). Moreover, these metabolites were significantly higher in the ESD and ESCC groups than in the HC group (**Table 2**). Also, creatinine and aminomalonic acid had ORs > 1 when ESCC vs. ESD (**Table 4**). These two substances were significantly increased in ESCC relative to ESD. In particular, aminomalonic acid increased with the progression of the ESCC condition. These results suggested that higher glyceric acid, oxalic acid, hexadecanoic acid, and 4-hydroxybutanoic acid plasma levels increase HC’s risk of being diagnosed as ESD. And higher creatinine and aminomalonic acid plasma level increase the risk of ESD being diagnosed as ESCC. Meanwhile, plasma alpha-tocopherol was significantly inversely associated with the risk of ESD and ESCC after adjusting for age and sex (OR<1) (**Table 4**). Lower plasma concentrations of cysteine were associated with a significantly increased risk of ESCC

**Table 4:** List of risk factors: ESD vs. HC, ESCC vs. ESD and N (1 + 2 + 3) vs. N0.

Groups	Risk factors	Adjusted OR	95% CI	P
ESD vs. HC	Glyceric acid	171317.41	951.97 - 30830384.91	0.002
	Oxalic acid	57715.64	347.82 - 9576957.08	0.001
	Hexadecanoic acid	237.48	14.43 - 3909.02	0.000
	4-Hydroxybutanoic acid	181.86	4.91 - 6733.25	0.026
	Cysteine	2.85E-03	4.30E-05 - 1.88E-01	0.001
	Alpha-Tocopherol	4.42E-07	6.49E-10 - 3.01E-04	0.000
ESCC vs. ESD	Creatinine	45.34	2.72 - 755.68	0.004
	Aminomalonic acid	13.61	1.36 - 136.44	0.013
	Alpha-Tocopherol	0.47	0.11 - 1.92	0.019
N (1 + 2 + 3) vs. N0	Aminomalonic acid	3.41	1.04 - 11.14	0.046
	Pyrophosphoric acid	4.97	1.03 - 24.00	0.040
	Uric acid	4.58	1.21 - 17.25	0.015

Relative to the group without lymphatic metastases, there were five differential metabolites in ESCC patients with lymphatic metastases, with decreased succinate and glyceric acid levels and increased aminomalonic acid, pyrophosphoric acid, and uric acid (**Supplementary Table 5**). Aminomalonic acid, pyrophosphoric acid, and uric acid had ORs > 1 and may be risk factors for developing lymphatic metastases in patients with ESCC (**Table 4**).

### Predictive Modeling

To construct effective diagnostic models, we applied logistic regression analysis using the data from the training set. First, binary logistic regression analysis and an optimized algorithm of the stepwise forward method (Wald) method were applied to establish the best model using the above differential metabolites. Eventually, the combination of five metabolites was defined as the ideal biomarker panel to discriminate ESCC and ESD from HC. These five metabolites are glycolic acid, oxalic acid, glyceric acid, malate and alpha-tocopherol.

**Figure 3:**
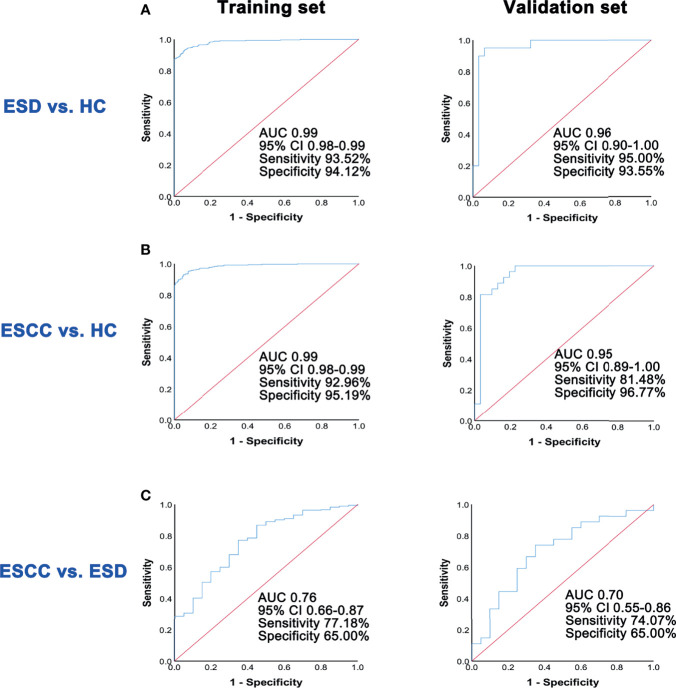
ROC analysis of predictive models in the training set and the validation set. **(A)** ROC analysis results of the prediction model in training set and validation set for ESD and HC groups. **(B)** ROC analysis results of the prediction model in training set and validation set for ESCC and HC groups. **(C)** ROC analysis results of the prediction model in training set and validation set for ESCC and ESD groups.

The diagnostic potential of these five metabolites was evaluated in both the training set and the validation set. To discriminate ESD from HC, the AUC value of the training set was 0.99 (95% CI: 0.98-0.99, Sensitivity = 93.52%, Specificity = 94.12%), whereas that of the validation set was 0.96 (95% CI: 0.90-1.00, Sensitivity = 95.00%, Specificity = 93.55%) ​(**Figure 3A**). To discriminate ESCC from HC, the AUC value of the training set was 0.99 (95% CI: 0.98-0.99, Sensitivity = 92.96%, Specificity = 95.19%), whereas that of the validation set was 0.95 (95% CI: 0.89-1.00, Sensitivity = 81.48%, Specificity = 96.77%) ​(**Figure 3B**).

Similarly, in the discrimination between ESCC and ESD, five differential metabolites, including glycine, nonanoic acid, aminomalonic acid, arachidonic acid, and alpha-tocopherol were selected using the logistic regression model. The AUC value of the training set was 0.76 (95% CI: 0.66-0.87, Sensitivity =77.18%, Specificity = 65.00%), whereas that of the validation set was 0.70 (95% CI: 0.55-0.86, Sensitivity = 74.07%, Specificity = 65.00%) ​(**Figure 3C**).

## Discussion

This study found that ESD and ESCC have similar metabolic phenotypes. From a metabolomic perspective, we suggested that ESD may be an early manifestation of ESCC, and prevention of ESD may be beneficial in preventing the development of ESCC. Meanwhile, as the disease of ESCC patients continued to worsen, their plasma levels of oxidative stress-related metabolites (alpha-tocopherol, cysteine, and aminomalonic acid) continued to change abnormally in ESCC patients at different stages. The development of esophageal cancer is associated with abnormal levels of oxidative stress. The use of antioxidants and regulating oxidative stress levels in the body may help prevent and control early-stage esophageal cancer (25–27).

Traditionally, alpha-tocopherol is considered the most active form of vitamin E in humans and is a powerful biological antioxidant. In the present study, lower plasma concentrations of alpha-tocopherol were associated with a significantly increased risk of ESCC. Previous large-scale intervention studies have shown that alpha-tocopherol deficiency is associated with the development of ESCC (28). Hui Yang et al. found that supplementation with alpha-tocopherol may prevent ESCC by modulating the PPAR γ-Akt signaling pathway and attenuating NF-κB activation and CXCR3-mediated inflammation without effect in the late stage of ESCC carcinogenesis (29, 30). Therefore, we believe that early supplementation with alpha-tocopherol may have a preventive effect on ESCC.

Cysteine plays an essential role in the metabolic rewiring of cancer cells, participating in glutathione synthesis, contributing to oxidative stress control; acting as a substrate for hydrogen sulfide production (H_2_S), stimulating cellular bioenergy; and as a carbon source for biomass and energy production. Gwen Murphy et al. found that higher serum concentrations of cysteine were associated with a significantly reduced risk of oesophageal squamous cell carcinomas (31). Moreover, the level of cysteine in tumor tissue of ESCC patients was significantly higher than that in adjacent tissue (32). Therefore, we hypothesize that tumor tissues of ESCC patients may increase the uptake of plasma cysteine to maintain oxidative stress homeostasis and meet bioenergy requirements in tumors.

We found that aminomalonic acid levels increased at various exacerbation stages in ESCC patients, which may be a risk factor for ESCC. However, aminomalonic acid has never been suggested to play a role in esophageal diseases. Previously, several studies have found that altered aminomalonic acid levels in blood were associated with colorectal cancer, abdominal aortic aneurysm and type 2 diabetes (33–35). Moreover, aminomalonic acid is closely associated with oxidative damage biomarkers (8-isopropanedioic acid and 8-OHdG), and its origin may be related to free radical-mediated protein oxidation (36). Therefore, the elevated aminomalonic acid levels in ESCC patients may be associated with impaired function, including esophageal, gastrointestinal and hepatic functions due to long-term poor diet.

Although alpha-tocopherol showed its potential in distinguishing HC from ESD (AUC= 0.92, sensitivity = 0%, specificity = 100%) and ESCC (AUC= 0.91, sensitivity = 0%, specificity = 100%). Its sensitivity was poor. To improve the diagnostic performance of alpha-tocopherol, we used a combined biosignature of glycolic acid, oxalic acid, glyceric acid, malate and alpha-tocopherol. This combination greatly improved the ability to differentiate between HC and ESD/ESCC (AUC>0.95) and had good sensitivity and specificity. Unfortunately, we did not find a good combination of metabolites and metabolites to distinguish ESD from ESCC in this study.

### Limitations

We collected patients with ESCC and performed a comprehensive analysis of their metabolic phenotypes and metabolic characteristics, but there are still some limitations. The major limitation of the present study is that it is a single-center study, and it is unclear whether the findings apply to other regions and populations. Although many metabolic changes associated with ESCC were identified in this study, further mechanistic studies are lacking. In the future, we will combine multiple centers, expand the sample size to validate our experimental results, and conduct mechanistic studies on the metabolic characteristics of ESCC.

## Conclusion

The development of ESCC is accompanied by persistent abnormal changes in oxidative stress in patients. Improving the body’s antioxidant capacity may help prevent the development of ESCC.

## Data Availability Statement

The raw data supporting the conclusions of this article will be made available by the authors, without undue reservation.

## Ethics Statement

The studies involving human participants were reviewed and approved by The ethics committee of the First Affiliated Hospital of Nanjing Medical University. The patients/participants provided their written informed consent to participate in this study.

## Author Contributions

WZ and JA were responsible for the concept of the study. GW and JA provided the GC/MS platform. WW collected the blood samples, recorded the medical history of the volunteers, and prepared the plasma samples. MY performed the untargeted metabolomics. MY and XY analyzed the data. MY and WW wrote the manuscript. All authors contributed to the article and approved the submitted version.

## Funding

This study was funded by the leading technology foundation research project of Jiangsu Province (BK20192005) and the National Natural Science Foundation of the People’s Republic of China (82173890&81773814). We thank all the investigators, study nurses, patients, and their family members.

## Conflict of Interest

The authors declare that the research was conducted in the absence of any commercial or financial relationships that could be construed as a potential conflict of interest.

## Publisher’s Note

All claims expressed in this article are solely those of the authors and do not necessarily represent those of their affiliated organizations, or those of the publisher, the editors and the reviewers. Any product that may be evaluated in this article, or claim that may be made by its manufacturer, is not guaranteed or endorsed by the publisher.
