# Supplementary material for: Plasma Metabolomics Reveals Diagnostic Biomarkers and Risk Factors for Esophageal Squamous Cell Carcinoma
Source: Front Oncol. 2022 Feb 7;12:829350. doi: 10.3389/fonc.2022.829350 (PMC8859148; doi:10.3389/fonc.2022.829350)
Supplement: Supplementary file 1 [file DataSheet_1.pdf]

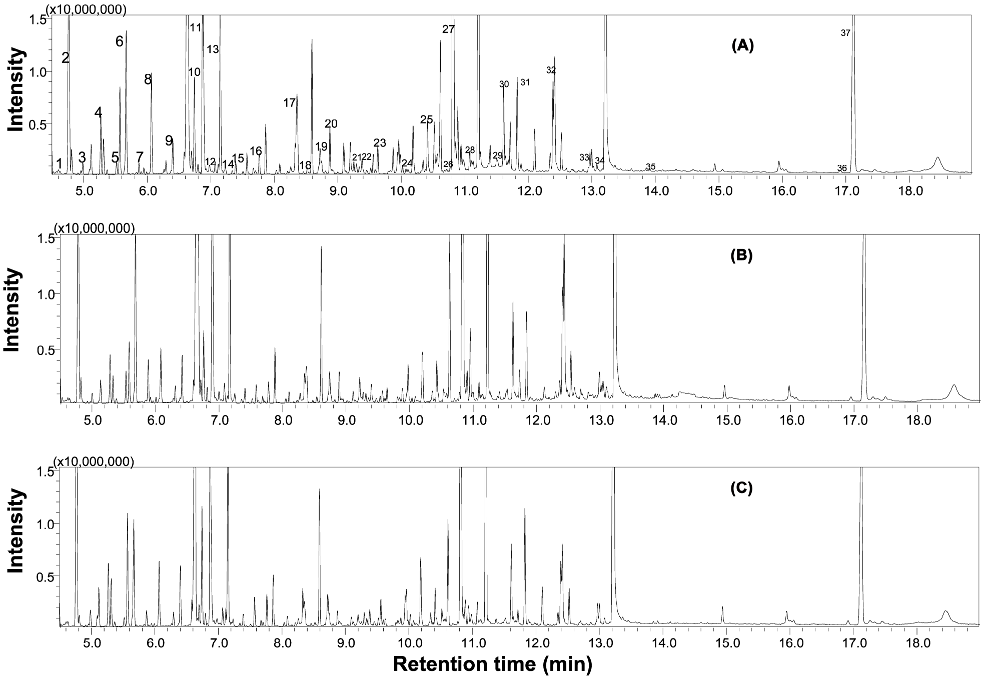

Supplementary Figure 1. Typical GC-MS chromatograms and identified molecules in the plasma from the ESCC (A), the ESD (B) and the HC (C).

The molecules were aligned as: 1, Pyruvate; 2, Lactate; 3, Glycolic acid; 4, Alanine; 5, Glycine; 6, Oxalic acid; 7, 3-Hydroxybutyric acid; 8, Urea; 9, Valine; 10, Serine; 11, Phosphate; 12, Leucine; 13, Proline; 14, Succinate; 15, Glyceric acid; 16, Threonine; 17, Aminomalonic acid; 18, Malate; 19, Aspartate; 20, Creatinine; 21, Glutamate; 22, Phenylalanine; 23, Asparagine; 24, Glutamine; 25, Citrate; 26, Fructose; 27, Glucose; 28, Hexadecanoic acid; 29, Palmitelaidic acid; 30, Palmitic acid; 31, Myo-Inositol; 32, Oleic acid; 33, Fructose-6-Phosphate ; 34, Arachidonic acid; 35, Monopalmitin; 36, Alpha-Tocopherol; 37, Cholesterol.

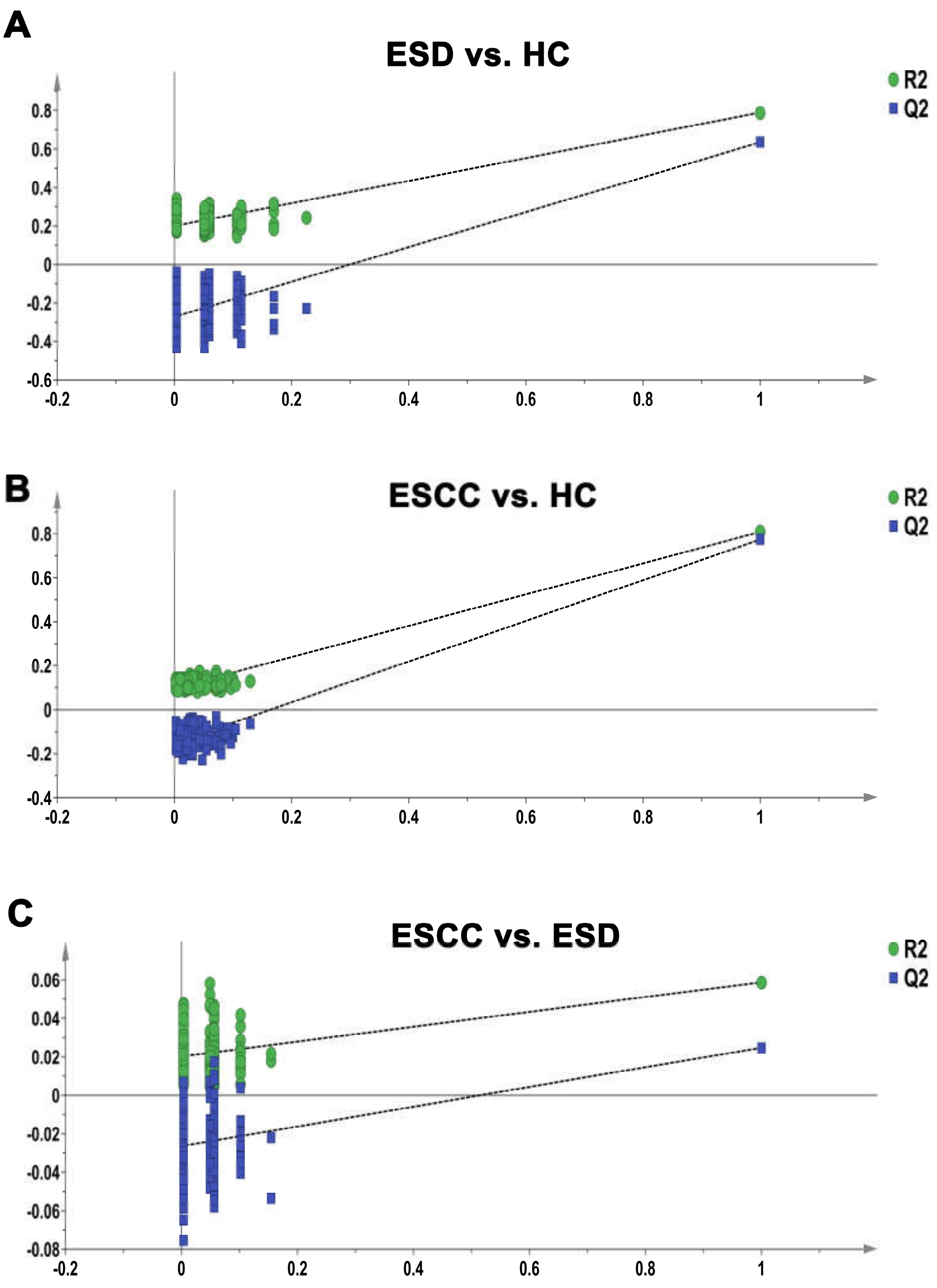

Supplementary Figure 2. Permutation test score plots. (A) Permutation test score plot between ESD and HC. (B) Permutation test score plot between ESCC and HC. (C) Permutation test score plot between ESCC and ESD.

Supplementary Table1. The identified endogenous compounds in plasma using GC/MS system.

Identification	Retention time (min)	Quant mass(m/z)
3-Hydroxybutyric acid-2TMS	5.885	191
3-hydroxypyridine	4.53	152
4-Hydroxybutanoic acid	6.595	233
Alanine	5.285	116
Alpha-Tocopherol	16.955	502
Aminomalonic acid	8.37	218
Arachidonic acid	13.1	117
Asparagine	9.61	231
Aspartate-3TMS	8.695	232
beta-Alanine	8.1	248
Cholesterol-TMS	17.15	458
Citrate	10.43	273
Creatinine	8.95	115
Cysteine	8.93	220
Cystine	12.745	218
Fructose	10.755	307
Fructose-6-Phosphate	12.97	315
Fumarate	7.53	245
Glucose	10.835	319
Glutamate-3TMS	9.31	246
Glutamine-3TMS	10.205	156
Glyceric acid	7.355	292
Glycine-2TMS	7.2	174
Glycine-TMS	5.495	102
Glycolic acid	4.975	177
Hexadecanoic acid	11.055	270
Isoleucine	7.09	158
Lactate	4.785	191
Leucine	6.905	158
Linoleic acid	12.405	337
Lysine	11	317
Malate-3TMS	8.475	233
Methionine	8.715	176
Monomethylphosphate	6.04	241
Monopalmitin	13.935	371
Myo-Inositol	11.84	318
Nonanoic acid	7.625	215
Oleic acid	12.42	202
Ornithine	10.455	142
Oxalic acid	5.685	220
Palmitelaidic acid	11.54	311
Palmitic acid	11.63	313

Phenylalanine	9.4	218
Phosphate	6.89	314
Proline	7.135	142
Pyroglutamate-TMS	8.74	156
Pyrophosphoric acid-4TMS	9.54	451
Pyruvate	4.65	174
Serine-2TMS	6.785	116
Serine-3TMS	7.59	204
Succinate	7.235	247
Threonine	7.8	218
Tyrosine	11.095	218
Urea-2TMS	6.64	205
Urea-3TMS	6.085	261
Uric acid	11.84	441
Valine	6.42	218

Supplementary Table 2. ROC analysis results of differential metabolites between ESD and HC.

Differential metabolites	AUROC	95% CI	Sensitivity	Specificity	Cut-off value
Alpha-tocopherol	0.92	0.86-0.98	0.00%	100.00%	6.50
Linoleic acid	0.89	0.79-0.99	0.00%	100.00%	6.44
Malate	0.89	0.73-1.00	15.38%	97.96%	4.20
Pyruvate	0.88	0.75-1.00	7.69%	98.64%	5.05
Lactate	0.87	0.72-1.00	15.38%	99.32%	6.60
Glutamine	0.86	0.77-0.96	100.00%	3.40%	5.83
Asparagine	0.85	0.75-0.95	100.00%	4.76%	4.16
Ornithine	0.83	0.72-0.95	100.00%	1.36%	4.51
Palmitic acid	0.83	0.70-0.96	0.00%	100.00%	6.94
Succinate	0.83	0.70-0.96	7.69%	99.32%	4.04
Aspartate	0.82	0.68-0.95	7.69%	100.00%	4.72
Pyrophosphoric acid	0.80	0.65-0.96	100.00%	0.68%	4.71
Alanine	0.80	0.67-0.92	100.00%	1.36%	5.94
Citrate	0.80	0.69-0.90	100.00%	1.36%	5.43
Oxalic acid	0.78	0.61-0.95	69.23%	84.35%	5.49
Fumarate	0.78	0.66-0.89	100.00%	8.84%	3.88
Hexadecanoic acid	0.75	0.58-0.91	61.54%	83.67%	3.37
Arachidonic acid	0.74	0.56-0.92	76.92%	65.99%	4.29
Valine	0.74	0.65-0.83	100.00%	6.12%	5.12
Uric acid	0.73	0.59-0.88	0.00%	100.00%	7.12
4-hydroxybutanoic acid	0.73	0.56-0.90	38.46%	97.28%	5.04
Phenylalanine	0.73	0.63-0.83	92.31%	17.01%	5.12
Cysteine	0.72	0.53-0.92	15.38%	89.12%	4.54
Glycine	0.71	0.60-0.81	100.00%	9.52%	3.76
Proline	0.70	0.52-0.88	7.69%	100.00%	6.25
Serine	0.70	0.56-0.84	100.00%	0.68%	5.12
Myo-inositol	0.70	0.54-0.86	0.00%	100.00%	6.26
Leucine	0.70	0.61-0.79	100.00%	10.20%	5.74
Lysine	0.68	0.56-0.81	100.00%	11.56%	4.31
Tyrosine	0.68	0.58-0.79	100.00%	8.16%	5.43
Glutamate	0.67	0.51-0.84	15.38%	93.20%	5.59
Glyceric acid	0.67	0.45-0.89	53.85%	95.92%	4.03
Monopalmitin	0.66	0.49-0.82	15.38%	88.44%	4.43
Nonanoic acid	0.64	0.49-0.79	61.54%	72.79%	3.71
Fructose	0.53	0.34-0.73	30.77%	82.31%	4.95

Supplementary Table 3. ROC analysis results of differential metabolites between ESCC and HC.

Differential metabolites	AUROC	95% CI	Sensitivity	Specificity	Cut-off value
Alpha-Tocopherol	0.91	0.88-0.94	0.00%	100.00%	6.50
Succinate	0.83	0.79-0.87	3.11%	98.64%	3.99
Malate	0.83	0.79-0.87	6.92%	97.28%	4.17
Asparagine	0.82	0.77-0.86	0.00%	100.00%	5.76
Linoleic acid	0.81	0.77-0.85	0.35%	100.00%	5.45
Glutamine	0.81	0.77-0.86	0.00%	100.00%	7.37
Monopalmitin	0.80	0.75-0.85	0.35%	100.00%	4.56
Oxalic acid	0.79	0.75-0.84	72.66%	78.91%	5.46
Hexadecanoic acid	0.79	0.75-0.83	66.78%	80.27%	3.34
Palmitic acid	0.77	0.72-0.81	0.69%	100.00%	5.95
Alanine	0.77	0.72-0.81	100.00%	0.68%	5.77
Pyroglutamate	0.75	0.70-0.80	15.57%	98.64%	5.75
Pyruvate	0.75	0.70-0.79	11.76%	96.60%	4.91
Glucose	0.74	0.69-0.79	75.78%	68.03%	5.94
Pyrophosphoric acid	0.73	0.68-0.78	100.00%	0.68%	4.61
Ornithine	0.72	0.67-0.78	0.35%	100.00%	5.56
Valine	0.72	0.67-0.78	100.00%	0.68%	4.95
Serine	0.72	0.67-0.77	0.35%	100.00%	5.82
Tyrosine	0.72	0.67-0.77	100.00%	0.68%	5.17
Aminomalonic acid	0.71	0.66-0.76	68.86%	65.31%	5.53
Cysteine	0.70	0.65-0.75	1.73%	100.00%	4.64
Methionine	0.70	0.65-0.76	0.35%	100.00%	5.03
Creatinine	0.70	0.65-0.75	60.90%	71.43%	4.59
Lactate	0.70	0.65-0.75	15.92%	96.60%	6.54
Citrate	0.69	0.64-0.74	3.46%	97.96%	5.84
Leucine	0.69	0.63-0.74	100.00%	0.68%	5.58
Fumarate	0.68	0.63-0.74	3.81%	99.32%	4.42
Proline	0.68	0.63-0.74	0.69%	100.00%	6.20
Phenylalanine	0.68	0.62-0.74	1.04%	99.32%	5.48
Myo-Inositol	0.68	0.63-0.73	0.00%	100.00%	6.26
Aspartate	0.68	0.63-0.73	11.76%	94.56%	4.57
Oleic acid	0.67	0.61-0.72	95.50%	5.44%	5.27
Glycolic acid	0.64	0.58-0.69	49.83%	76.19%	3.92
Lysine	0.63	0.57-0.69	97.23%	5.44%	4.18
Palmitelaidic acid	0.62	0.56-0.67	12.80%	93.88%	4.42
Threonine	0.61	0.55-0.67	1.38%	100.00%	5.41
Uric acid	0.60	0.55-0.66	1.38%	100.00%	6.13
Glyceric acid	0.60	0.55-0.66	34.95%	95.92%	4.02
3-Hydroxybutyric acid	0.60	0.55-0.66	9.00%	95.92%	5.52
Glutamate	0.60	0.54-0.65	4.15%	98.64%	5.64
Monomethylphosphate	0.59	0.53-0.65	100.00%	0.68%	3.69
Urea	0.57	0.51-0.63	26.30%	86.39%	5.63

Glycine	0.57	0.50-0.63	76.12%	27.89%	3.90
Cholesterol	0.56	0.51-0.62	2.77%	97.96%	5.97
Fructose-6-Phosphate	0.56	0.51-0.62	97.92%	4.08%	3.56
4-Hydroxybutanoic acid	0.56	0.50-0.62	64.01%	54.42%	4.82

Supplementary Table 4. ROC analysis results of differential metabolites between ESCC and ESD.

Differential metabolites	AUROC	95% CI	Sensitivity	Specificity	Cut-off value
Arachidonic acid	0.71	0.53-0.88	92.68%	15.00%	3.99
Glycine	0.69	0.56-0.81	33.24%	95.00%	4.16
4-hydroxybutanoic acid	0.67	0.50-0.84	0.28%	100.00%	5.25
Aminomalonic acid	0.66	0.49-0.84	75.77%	60.00%	5.46
Creatinine	0.66	0.50-0.82	69.01%	65.00%	4.54
Fructose	0.63	0.49-0.78	75.49%	65.00%	4.12
Uric acid	0.63	0.47-0.78	83.38%	45.00%	5.59
Nonanoic acid	0.62	0.46-0.79	10.14%	95.00%	4.22
Alpha-tocopherol	0.54	0.40-0.69	4.51%	100.00%	5.11

Supplementary Table 5. Differential metabolites between ESCC without lymphatic metastases (N0) and ESCC with lymphatic metastases(N(1+2+3)).

Metabolites	N0 (n=207)	N1+N2+N3 (n=139)	Trend	P
Succinate	3.67±0.18	3.63±0.14	down	0.006
Glyceric acid	4.02±0.19	3.98±0.15	down	0.045
Aminomalonic acid	5.55±0.20	5.59±0.17	up	0.046
Pyrophosphoric acid	4.97±0.14	5.00±0.14	up	0.040
Uric acid	5.73±0.18	5.77±0.17	up	0.015
